# Associations Between 25-Hydroxyvitamin D, Kidney Function, and Insulin Resistance Among Adults in the United States of America

**DOI:** 10.3389/fnut.2021.716878

**Published:** 2022-02-15

**Authors:** Jiwen Geng, Yuxuan Qiu, Yupei Li, Jiameng Li, Ruoxi Liao, Heyue Du, Luojia Jiang, Liya Wang, Zheng Qin, Qinbo Yang, Qiao Yu, Zhuyun Zhang, Baihai Su

**Affiliations:** ^1^Department of Nephrology, West China Hospital, Sichuan University, Chengdu, China; ^2^Department of Ultrasound, West China Hospital, Sichuan University, Chengdu, China; ^3^Institute for Disaster Management and Reconstruction, Sichuan University, Chengdu, China; ^4^The First People's Hospital of Shuangliu District, Chengdu, China

**Keywords:** 25-hydroxyvitamin D, kidney function, insulin resistance, metabolic syndrome, glomerular filtration rate

## Abstract

**Background:**

Although many molecular studies have tried to explore the relationship between vitamin D metabolism and kidney function, the association between 25-hydroxyvitamin D [25(OH)D] and kidney function is still controversial. Previous studies reported that low vitamin D status and decreased kidney function were associated with insulin resistance (IR). However, neither of them was confirmed by large population-based studies. This study evaluated the associations between 25(OH)D and kidney function and the associations between both of them and IR among adults in the United States of America (USA).

**Methods:**

We analyzed 36,523 adults from the National Health and Nutrition Examination Survey (NHANES) (2001–2014). Kidney function was assessed by the estimated glomerular filtration rate (eGFR), and IR was assessed by homeostasis model assessment (HOMA-IR). All data were survey-weighted, and corresponding linear regression models were performed to examine the associations.

**Results:**

The mean serum 25(OH)D levels were found to be increased in participants with decreased kidney function (eGFR <90 ml/min/1.73 m^2^), and each unit of decreased serum 25(OH)D concentrations predicted 0.453 ml/min/1.73 m^2^ (95% CI: 0.426 to 0.480, *p* < 0.0001) higher eGFR. In addition, each unit of decreased eGFR was associated with 0.007 higher HOMA-IR, while each unit of decreased 25(OH)D concentrations led to 0.025 higher HOMA-IR.

**Conclusions:**

Serum 25-hydroxyvitamin D concentrations were negatively associated with kidney function. IR appears in the early stage of kidney dysfunction, and both serum 25(OH)D concentrations and kidney function are negatively associated with IR. Clinicians should maintain appropriate serum 25(OH)D concentrations and doses of vitamin D supplements for different populations. The underlying mechanism of these associations still needs more research, especially the negative association between serum 25(OH)D concentrations and kidney function.

## Introduction

Humans can acquire vitamin D from sunlight exposure, diet, and dietary supplements (1). The main circulating form of vitamin D is 25-hydroxyvitamin D [25(OH)D], which is also the measure of vitamin D that the body stores (2). With the enzyme 1-a hydroxylase (CYP27B1), 25-hydroxyvitamin D [25(OH)D] can be converted to its more active form, 1,25-dihydroxyvitamin D [1,25(OH)2D]. 1,25-dihydroxyvitamin D has endocrine effects in the gut, parathyroid gland, kidneys, and bones (3). Abnormal kidney function can cause a decrease in the ability of the kidney to produce 1,25(OH)2D (4). Disruption of vitamin D metabolism can result in calcium, phosphorus, and bone metabolism disorders and even secondary hyperparathyroidism (SHPT) (5). However, vitamin D treatment for patients with kidney diseases has always been controversial. Although many studies support the use of vitamin D in patients with kidney diseases, especially for patients with SHPT (2, 3, 6), the association between 25(OH)D and estimated glomerular filtration rate (eGFR) is also confusing. A previous study showed that even though 25(OH)D levels were sufficient, they did not seem to have the ability to protect kidney function (7). In addition, with increasing 25(OH)D levels, kidney function represented by eGFR significantly decreased (8).

Insulin resistance (IR) is characterized by a reduced action of insulin despite increased insulin concentrations (hyperinsulinemia) (9, 10). IR may cause a series of metabolic syndromes (11). IR is not limited to specific etiologies of kidney disease, and it is common in patients with chronic kidney disease (CKD) (12). With the decrease in glomerular filtration rate, IR occurs more frequently and is almost universal in patients with end-stage kidney failure (ESKF) (13). In addition, IR in patients with CKD was associated with poor functional outcomes and risk factors that contribute to cardiovascular disease (CVD) (13, 14). Much evidence supports that low vitamin D status is associated with IR (15–17). Vitamin D deficiency can accelerate the progression of IR, and vitamin D supplement can reduce IR and the related pathologies (15). However, most of the previous studies were based on clinic-based samples, and few focused on large samples at the population level. In addition to affecting blood glucose, vitamin D is thought to be a risk factor for metabolic syndrome (18). Many studies have supported the link between vitamin D and IR, suggesting that vitamin D could directly improve insulin sensitivity and indirectly improve pancreatic β-cell function and IR by reducing inflammation (19). However, conclusive pieces of evidence from more studies are still needed.

Therefore, we used the National Health and Nutrition Examination Survey (NHANES) data representing the US population, which included more than 35,000 samples, aimed to examine the relationship among vitamin D levels, kidney function, and IR at the population level.

## Methods

### Data Source and Participants

We conducted a retrospective analysis of a cohort in the US population. The NHANES is a periodic survey performed by the National Center for Health Statistics (NCHS) with informed consent from every participant. Therefore, there was no need for any ethical consent in this study. NHANES includes extensive demographic data, physical examinations, laboratory tests, health-related questionnaires, and lists of prescription medications. Data from NHANES (2001–2014) is the only continuous collection providing information on laboratory tests of 25(OH)D and variables to calculate eGFR, fasting glucose, and insulin. The NHANES cycles (2000–2014) covered 72,126 participants, of which 15,889 individuals without 25(OH)D data, 11,585 participants without serum creatinine data, and 130 with an eGFR ≤ 15 ml/min/1.73 m^2^ or self-reported receiving dialysis were excluded. In addition, 7,999 children or adolescents (age ≤ 20 years) were also excluded. Finally, 36,523 individuals were included in the final analysis (Supplementary Figure 1).

### Measures of Serum 25(OH)D Concentration

Serum 25(OH)D concentration was tested using the principle that utilizes ultra-high performance liquid chromatography–tandem mass spectrometry (UHPLC-MS/MS) for quantitative detection.

### Measures of Kidney Function

The main biochemistry outcomes were covered by standard biochemistry profiles in the NHANES. Blood samples collected during the examination were centrifuged, aliquoted, frozen to −70°C on site, and shipped on dry ice to central laboratories, where they were stored at −70°C until analysis. The LX20 modular chemistry side uses the Jaffe rate method (kinetic alkaline picrate) to determine the concentration of creatinine in serum, plasma, or urine (20).

The eGFR was calculated according to the following equation: GFR = 141 ^*^ min(Scr/k, 1)^α^^*^ max(Scr/k, 1)^−1.209^^*^ 0.993^Age^^*^ 1.018 [if female] ^*^ 1.159 [if black], where Scr is serum creatinine, k is 0.7 for females and 0.9 for males, α is −0.329 for females and −0.411 for males, min indicates the minimum of Scr/k or 1, and max indicates the maximum of Scr/k or 1 (21).

The estimated GFR was reported in mL/min/1.73 m^2^ and grouped as normal (≥90), mild (60–90), moderate (30–59), and severe (15–29). Participants with eGFR <15 and self-reported receiving dialysis on the questionnaire “Kidney Conditions - Urology” were excluded.

### Measures of Insulin Resistance

Insulin levels were tested by the Merocodia insulin enzyme-linked immunosorbent assay, which is a two-site enzyme immunoassay utilizing the direct sandwich technique. Plasma glucose was measured by a modified hexokinase enzymatic method. IR was assessed by homeostatic model assessment (HOMA) (22). HOMA-IR was calculated using fasting glucose and insulin measurements as HOMA-IR = fasting insulin (mU/ml) ^*^ fasting glucose (mmol/l)/22.5. The fasting glucose value in mg/dL was converted to mmol/L by multiplying by 0.05551 (rounded to 3 decimals).

### Covariables

In the home interview, information was collected on a wide range of variables, including age, sex, ethnicity, smoking history, and drinking history. Information on the frequency of vitamin D supplements in the previous month was also collected. BMI was calculated as weight in kilograms divided by the square of height in meters. Blood pressure was measured at the mobile examination centers by physicians with mercury sphygmomanometers using a standard protocol. Current knowledge of heart disease, stroke, and diabetes was collected, and hypertension was defined as both told-to-have hypertension and tested systolic ≥140 mmHg or diastolic ≥90 mmHg.

### Missing Covariables

Addresses for 11% of the participants could not be geocoded and contributed to missing data in cross-sectional analyses. As such, 10 multiple imputations using fully conditional specifications were used to address potential biases arising from item non-response.

### Statistical Analysis

All statistical analyses were conducted according to CDC guidelines (https://wwwn.cdc.gov/nchs/nhanes/tutorials/default.aspx). Sample weight was taken into consideration and assigned to each participant (23). Continuous variables were presented as the mean ± standard error (se). Categorical variables were presented as frequencies or percentages. Weighted ANOVA (for continuous variables) or weighted chi-square test (for categorical variables) were used to calculate the differences among different groups.

The association between 25(OH)D and eGFR first employed weighted univariate and weighted multivariate line regression models. Two models were constructed and used in our analyses. The unadjusted and adjusted models were based on sex, age, ethnicity, ratio of family income to poverty, education level, BMI, drinking history, smoking history, diabetes, heart disease, stroke, hypertension, protein intake, vitamin D supplements, and serum albumin. To further address the non-linearity of 25(OH)D and eGFR, we conducted smooth curve fitting (penalized spline method) and weighted generalized additive model. Weighted continuous covariables were then converted into categorical variables according to their clinical cut points or quartiles and used to perform an interaction test. To ensure the robustness of the data analysis, we conducted the following sensitivity analysis. First, 25(OH)D was included as a categorical variable by quartiles, and testing for linear trends was performed. One purpose was to verify the results of 25(OH)D as a continuous variable, while another was to determine whether there was a non-linear relationship. The associations between eGFR, 25-hydroxyvitamin D and HOMA-IR were performed in a similar way as before, and the model adjusted not only the covariables above but also the addition of total cholesterol, high-density lipoprotein (HDL), low-density lipoprotein (LDL) and triglycerides. Furthermore, the interaction effect of eGFR and 25(OH)D was assessed by applying a weighted generalized additive model. Value of *p* < 0.05 was considered of significant difference. All analyses were conducted using StataSE 15.1 (StataCorp LLC, College Station).

## Results

### 25(OH)D Levels According to Demographic, Lifestyle, and Cardiometabolic Disease Variables

A total of 36,523 participants were included in the final analysis, 48.56% of whom were males. The Mean and mean difference in serum 25-hydroxyvitamin D (ng/ml) by level of demographic variables, lifestyle variables influencing vitamin D status and eGFR category are shown in Table 1. The percentages of normal kidney function and mildly, moderately, and severely decreased kidney functions were 60.93, 30.43, 7.96, and 0.68%, respectively. Mean concentrations of serum 25(OH)D were found to be higher in females (*p* < 0.0001), older people (*p* < 0.0001), people with lower BMI (P <0.0001), higher income ratio (*p* < 0.0001), higher education level (*p* < 0.0001), and more vigorous physical activity (*p* < 0.0001). In addition, the level of 25(OH)D showed significant ethnic differences (*p* < 0.0001)—it was highest in non-Hispanic whites and lowest in non-Hispanic blacks. In terms of lifestyle variables, mean serum vitamin D levels appeared to be increased with more daily doses of vitamin D supplements (*p* < 0.0001), current drinking habits (*p* < 0.0001) and former smoking history (*p* < 0.0001).

**Table 1:** Mean (s.e.) and mean difference (s.e.) in serum 25-hydroxyvitamin D (ng/ml) by level of demographic variables, lifestyle variables influencing vitamin D status, and eGFR category.

**Variable**	***N***	**Mean (s.e.)**	**Mean difference (s.e.)**	***P*-value**
Gender				<0.0001
Males	17,734	24.06 (0.07)	0	–
Females	18,789	24.76 (0.08)	0.69 (0.11)	<0.0001
Ages				<0.0001
20–29	8,613	22.81 (0.11)	0	–
30–39	5,775	23.85 (0.13)	1.04 (0.17)	<0.0001
40–49	5,860	23.68 (0.12)	0.87 (0.17)	<0.0001
50–59	5,005	24.69 (0.14)	1.88 (0.18)	<0.0001
60–69	5,282	25.24 (0.15)	2.43 (0.18)	<0.0001
70–79	3,623	26.80 (0.18)	3.99 (0.20)	<0.0001
≥80	2,365	27.50 (0.23)	4.69 (0.23)	<0.0001
Ethnicity				<0.0001
Non-hispanic white	16,978	28.55 (0.08)	0	–
Mexican American	6,556	21.58 (0.09)	−6.97 (0.14)	<0.0001
Other hispanic	2,718	23.80 (0.17)	−4.75 (0.19)	<0.0001
Non-hispanic black	7,503	18.17 (0.10)	−10.38 (0.13)	<0.0001
Other race including multi-racial	2,768	23.38 (0.19)	−5.17 (0.19)	<0.0001
Ratio of family income to poverty				<0.0001
<1	7,368	22.45 (0.11)	0	–
1–3.5	16,170	23.97 (0.08)	1.52 (0.14)	<0.0001
≥3.5	10,345	26.68 (0.10)	4.23 (0.15)	<0.0001
Education level				<0.0001
<9th Grade	4,036	22.85 (0.14)	0	–
9–11th grade (includes 12th grade with no diploma)	5,149	22.99 (0.14)	0.14 (0.21)	1
High school grad/GED or Equivalent	7,880	24.60 (0.12)	1.75 (0.20)	<0.0001
Some college or AA degree	9,566	24.65 (0.11)	1.8 (0.19)	<0.0001
College graduate or above	7,257	26.83 (0.12)	3.98 (0.20)	<0.0001
BMI				<0.0001
≤ 23.1	7,161	25.99 (0.13)	0	–
23.2–26.1	7,163	26.10 (0.12)	0.12 (0.17)	1
26.2–29.0	7,162	25.04 (0.12)	−0.94 (0.17)	<0.0001
29.1–33.1	7,158	23.8 (0.11)	−2.18 (0.17)	<0.0001
≥33.2	7,192	21.4 (0.11)	−4.59 (0.17)	<0.0001
Drink				<0.0001
Never	4,679	23.44 (0.15)	0	–
Former	4,629	23.67 (0.15)	0.24 (0.21)	0.7877
Current	22,363	25.18 (0.07)	1.74 (0.16)	<0.0001
Smoke				<0.0001
Never	18,399	24.38 (0.07)	0	–
Former	8,491	26.17 (0.11)	1.79 (0.13)	<0.0001
Current	7,309	23.40 (0.12)	−0.98 (0.14)	<0.0001
Vitamin D supplements (IU/day)				<0.0001
None	27,849	21.12 (0.12)	0	–
>0 to <200	1,505	22.03 (0.33)	0.91 (0.39)	0.1254
200 to <400	743	23.07 (0.25)	1.95 (0.29)	<0.0001
Physical activity (times/month)				<0.0001
Vigorous ≥12	1,952	25.35 (0.26)	4.12 (0.26)	<0.0001
Vigorous 1–11	3,566	26.53 (0.19)	5.3 (0.21)	<0.0001
Moderate ≥12	12,450	25.80 (0.09)	4.56 (0.15)	<0.0001
Moderate 1–11	11,285	24.06 (0.09)	2.82 (0.16)	<0.0001
Inactive	6,583	21.24 (0.12)	0	–
Heart disease				0.7017
Yes	2,526	24.72 (0.21)	0	–
No	31,266	24.63 (0.06)	−0.08 (0.21)	0.7017
Stroke				0.8556
Yes	1,235	24.69 (0.31)	0	–
No	32,649	24.64 (0.06)	−0.06 (0.32)	0.8556
Hypertension				0.0004
Yes	6,369	24.09 (0.13)	0	–
No	28,650	24.60 (0.06)	0.51 (0.14)	0.0004
Diabetes				<0.0001
Yes	3,834	23.7 (0.17)	−0.8 (0.17)	<0.0001
No	31,994	24.49 (0,06)	0	–
≥400	5,739	25.29 (0.06)	4.17 (0.15)	<0.0001
eGFR category (ml/min/1.73 m^2^)				<0.0001
Normal (≥90)	22,253	22.99 (0.06)	0	–
Mild (60–90)	11,113	26.47 (0.10)	3.48 (0.12)	<0.0001
Moderate (30–59)	2,908	27.29 (0.21)	4.3 (0.20)	<0.0001
Severe (15–29)	249	27.04 (0.90)	4.05 (0.64)	<0.0001

*BMI, body mass index; eGFR, estimated glomerular filtration rate; s.e., standard error*.

### Association Between 25(OH)D Concentrations and Kidney Function

Compared to participants with normal kidney function (eGFR ≥90 ml/min/1.73 m^2^), the mean serum 25(OH)D levels were higher in participants with decreased kidney function (eGFR <90 ml/min/1.73 m^2^), and it was highest in participants with moderately decreased kidney function (eGFR = 30–59 ml/min/1.73 m^2^) (Table 1).

The association between 25(OH)D concentrations and kidney function is shown in Table 2. Models were adjusted for sex, age, ethnicity, ratio of family income to poverty, education level, BMI, drinking history, smoking history, vitamin D supplements, physical activity, protein intake, serum albumin, heart disease, stroke, hypertension, and diabetes. With potential confounders adjusted, the eGFR of participants in the first quartile of serum 25(OH)D concentrations was 12.201 ml/min/1.73 m^2^ higher than those in the fourth quartile. In addition, each unit of decreased serum 25(OH)D concentrations predicted 0.453 ml/min/1.73 m^2^ (95% CI: 0.426, 0.48, *p* < 0.0001) higher eGFR. In addition, it suggested the protective effect of low vitamin D levels on kidney function.

**Table 2:** Association between vitamin D levels and kidney function.

**Serum 25-hydroxyvitamin D quartile**	**Unadjusted model**				**Adjusted model^b^**			
	**β^a^ (95% CI)**	***P*-value**	**OR (95%CI)**	***P*-value**	**β (95% CI)**	***P*-value**	**OR (95%CI)**	***P*-value**
Continuous	0.513 (0.488, 0.537)	<0.0001	0.965 (0.963, 0.967)	<0.0001	0.453 (0.426, 0.480)	<0.0001	0.967 (0.964, 0.969)	<0.0001
<16.9	13.806 (13.098, 14.514)	<0.0001	0.382 (0.359, 0.406)	<0.0001	12.201 (11.411, 12.99)	<0.0001	0.402 (0.374, 0.431)	<0.0001
16.9–23.5	9.476 (8.781, 10.171)	<0.0001	0.501 (0.473, 0.531)	<0.0001	8.103 (7.342, 8.864)	<0.0001	0.534 (0.500, 0.571)	<0.0001
23.5–30.2	5.678 (4.967, 6.389)	<0.0001	0.649 (0.612, 0.689)	<0.0001	4.795 (4.025, 5.564)	<0.0001	0.677 (0.633, 0.724)	<0.0001
>30.2	Ref.		Ref.		Ref.		Ref.	
*P* for trend	4.531 (4.307, 4.755)	<0.0001	0.728 (0.714, 0.742)	<0.0001	4.000 (3.752, 4.249)	<0.0001	0.741 (0.725, 0.758)	<0.0001

*Serum 25-hydroxyvitamin D was converted from a continuous variable to a categorical variable (quartiles), which revealed the change of eGFR with the decrease of vitamin D level*.

a *β: effect sizes*.

b *Adjusted Model: adjusted for gender, ages, ethnicity, ratio of family income to poverty, education level, BMI, drinking, smoking, vitamin D supplements, physical activity, protein intake, serum albumin, heart disease, stroke, hypertension, diabetes*.

*eGFR, estimated glomerular filtration rate; 95% CI: 95% Confidence interval; OR, odds ratio*.

### Mean Levels of the Components of Metabolic Syndrome

Metabolic syndrome is composed of abdominal obesity, blood pressure, high-density lipoprotein cholesterol, triglycerides, and fasting glucose (24). Mean levels of the components of metabolic syndrome were evaluated by eGFR category, and the serum 25(OH)D quartile is shown in Table 3.

**Table 3:** Mean (s.e.) levels of the components of metabolic syndrome.

**Variable**	**Waist circumference (cm)**	**BMI**	**Blood pressure (mmHg)**		**Cholesterol (mg/dL)**			**Triglycerides (mg/dL)**	**Blood glucose (mg/dL)**
			**Systolic**	**Diastolic**	**Total**	**HDL**	**LDL**		
Identification^a^	Male :>102 Female: >88	–	≥135	≥85	–	Male: <40 Female: <50	–	≥150	≥110
eGFR category (ml/min/1.73 m^2^)									
Normal (≥90)	95.85 (0.11)	28.39 (0.05)	118.67 (0.11)	69.20 (0.09)	192.29 (0.29)	52.80 (0.11)	112.69 (0.34)	130.93 (1.19)	103.06 (0.32)
Mild (60–90)	100.05 (0.14)	28.84 (0.06)	128.65 (0.19)	70.68 (0.13)	200.40 (0.40)	53.59 (0.15)	118.46 (0.51)	139.76 (1.56)	109.79 (0.49)
Moderate (30–59)	102.48 (0.29)	29.10 (0.12)	136.03 (0.44)	64.17 (0.32)	192.60 (0.84)	52.58 (0.30)	108.11 (1.08)	147.90 (2.51)	117.39 (1.09)
Severe (15–29)	104.91 (0.99)	30.14 (0.45)	139.83 (1.86)	60.26 (1.24)	181.72 (2.83)	49.33 (1.04)	101.56 (3.47)	162.68 (9.33)	125.21 (4.87)
*P*-value	<0.0001	<0.0001	<0.0001	<0.0001	<0.0001	<0.0001	<0.0001	<0.0001	<0.0001
25-hydroxyvitamin D quartile (ng/ml)									
<16.9	99.71 (0.20)	30.19 (0.09)	124.49 (0.22)	69.90 (0.15)	191.67 (0.47)	52.27 (0.17)	112.10 (0.57)	132.83 (2.39)	109.20 (0.64)
16.9–23.5	98.48 (0.17)	28.96 (0.07)	122.93 (0.19)	69.40 (0.14)	194.16 (0.44)	51.03 (0.15)	114.50 (0.53)	139.26 (1.67)	108.01 (0.57)
23.5–30.2	97.48 (0.17)	28.18 (0.07)	122.93 (0.20)	69.31 (0.14)	195.70 (0.45)	52.44 (0.17)	114.66 (0.56)	137.89 (1.63)	105.43 (0.47)
>30.2	95.17 (0.16)	27.11 (0.06)	122.66 (0.20)	68.21 (0.14)	197.22 (0.44)	56.19 (0.17)	114.54 (0.53)	130.10 (1.38)	102.55 (0.39)
*P*-value	<0.0001	<0.0001	<0.0001	<0.0001	<0.0001	<0.0001	0.002	0.0005	<0.0001

*BMI, body mass index; eGFR, estimated glomerular filtration rate; HDL, high-density lipoprotein; LDL, low density lipoprotein*.

a *Definition of metabolic syndrome proposed by the National Cholesterol Education Program's Adult Treatment Panel III report (ATP III)*.

Participants with severely decreased kidney function (eGFR = 15–29 ml/min/1.73 m^2^) had higher waist circumference, BMI, systolic blood pressure, triglycerides, and blood glucose values. In addition, those participants had lower diastolic blood pressure and cholesterol, including total cholesterol, high-density lipoprotein (HDL), and low-density lipoprotein (LDL) cholesterol. Participants in the first quartile of serum 25(OH)D concentrations seemed to have higher waist circumference, BMI, and blood glucose values.

### Association Between Kidney Function and IR

The mean fasting insulin and HOMA-IR were the highest in participants with severely decreased kidney function (eGFR = 15–29 ml/min/1.73 m^2^). IR appeared in the early stage of kidney dysfunction (Table 4).

**Table 4:** Mean (s.e.) levels of fasting insulin levels, and IR.

	**Fasting insulin (uU/mL)**		**HOMA-IR**	
	**Mean (s.e.)**	***P*-value**	**Mean (s.e.)**	***P*-value**
eGFR category (ml/min/1.73 m^2^)		<0.0001		<0.0001
Normal (≥90)	13.30(0.14)	–	2.27(0.02)	–
Mild (60–90)	13.13(0.20)	1	2.37(0.03)	0.0163
Moderate (30–59)	15.93(0.77)	<0.0001	2.81(0.08)	<0.0001
Severe (15–29)	16.46(1.56)	0.2083	3.02(0.23)	0.0002
Serum 25-hydroxyvitamin D quartile (ng/ml)		<0.0001		<0.0001
<16.9	15.26(0.23)	–	2.60(0.03)	–
16.9–23.5	14.50(0.26)	0.6496	2.51(0.03)	0.1440
23.5–30.2	12.93(0.28)	<0.0001	2.26(0.03)	<0.0001
>30.2	11.25(0.18)	<0.0001	2.02(0.02)	<0.0001

*eGFR, estimated glomerular filtration rate; IR, insulin resistance; HOMA-IR, Homeostasis model assessment of insulin resistance*.

The association between kidney function and IR is shown in Table 5. Kidney function was negatively associated with IR. After potential confounder adjustment, including sex, age, ethnicity, ratio of family income to poverty, education level, BMI, alcohol consumption, smoking history, diabetes, heart disease, stroke, hypertension, protein intake, serum albumin, vitamin D supplements, physical activity, total cholesterol, HDL cholesterol, LDL cholesterol, and triglycerides, the association was stable. Participants with severely decreased kidney function (eGFR = 15–29 ml/min/1.73 m^2^) had 0.802 higher HOMA-IR than participants with normal kidney function (eGFR ≥90 ml/min/1.73 m^2^), and each unit of decreased eGFR was associated with 0.007 higher HOMA-IR.

**Table 5:** Association between kidney function and IR and association between 25-hydroxyvitamin D concentrations and IR.

**Variable**	**Unadjusted model**		**Adjusted model^b^**	
	**β^a^ (95% CI)**	***P*-value**	**β (95% CI)**	***P*-value**
eGFR category (ml/min/1.73 m^2^)				
Continuous	0.008 (0.007, 0.009)	<0.0001	0.007 (0.006, 0.008)	<0.0001
Normal (≥90)	Ref.		Ref.	
Mild (60–90)	0.189 (0.124, 0.253)	<0.0001	0.165 (0.098, 0.231)	<0.0001
Moderate (30–59)	0.633 (0.524, 0.742)	<0.0001	0.446 (0.332, 0.560)	<0.0001
Severe (15–29)	0.805 (0.452, 1.158)	<0.0001	0.802 (0.408, 1.197)	<0.0001
*P* for trend	0.264 (0.221, 0.307)	<0.0001	0.206 (0.16, 0.251)	<0.0001
Serum 25-hydroxyvitamin D quartile (ng/ml)				
Continuous	0.026 (0.024, 0.029)	<0.0001	0.025 (0.022, 0.028)	<0.0001
<16.9	0.645 (0.563, 0.726)	<0.0001	0.644 (0.557, 0.731)	<0.0001
16.9–23.5	0.537 (0.458, 0.616)	<0.0001	0.481 (0.398, 0.563)	<0.0001
23.5–30.2	0.274 (0.193, 0.355)	<0.0001	0.245 (0.162, 0.329)	<0.0001
>30.2	Ref.		Ref.	
*P* for trend	0.224 (0.198, 0.250)	<0.0001	0.22 (0.193, 0.248)	<0.0001

*Serum 25-hydroxyvitamin D was converted from a continuous variable to a categorical variable (quartiles), which revealed the change of eGFR with the decrease of vitamin D level*.

a *β: effect sizes*.

b *Adjusted Model: adjusted for gender, ages, ethnicity, ratio of family income to poverty, education level, BMI, drinking, smoking, diabetes, heart disease, stroke, hypertension, protein intake, serum albumin, vitamin D supplements, physical activity, total cholesterol, high-density lipoprotein (HDL) cholesterol, low-density lipoprotein (LDL) cholesterol, and triglycerides*.

*eGFR, estimated glomerular filtration rate; IR, insulin resistance; 95% CI: 95% Confidence interval*.

### Association Between 25(OH)D Concentrations and IR

The mean fasting insulin and HOMA-IR levels were highest in participants in the first quartile of serum 25(OH)D concentrations (Table 4).

Table 5 suggests that vitamin D had an inverse association with IR, and the association was in accordance with both the unadjusted and adjusted models. The HOMA-IR scores of participants in the first quartile of serum 25(OH)D concentrations were 0.644 higher than those of participants in the fourth quartile. Each unit of decreased serum 25(OH)D concentrations would lead to 0.025 higher HOMA-IR scores.

## Discussion

Vitamin D status is customarily defined as deficient, insufficient, and sufficient when the serum 25(OH)D concentrations are <20 ng/ml, 20–29.9 ng/ml, and more than 30 ng/ml, respectively (1, 7, 25). The high prevalence of vitamin D deficiency (VDD) and vitamin D insufficiency (VDI) was shown in past NHANES and Korean NHANES (KNHANES) studies (26, 27). Our research presented similar results, which suggested that VDD and VDI could be public nutrition problems that need to be given more attention. Due to kidney functional abnormalities, the metabolism and utilization of vitamin D are influenced (3, 4). With the support of previous research, VDD is thought to be worse in patients with CKD (28). However, our research presented different results: the mean 25(OH)D concentrations were increased in participants with decreased kidney function (eGFR <90 ml/min/1.73 m^2^) compared with participants with normal kidney function (eGFR ≥90 ml/min/1.73 m^2^). The reason could be that patients with CKD are more likely to take vitamin D supplements and calcitriol than those with normal kidney function.

Many studies have suggested the use of vitamin D in patients with CKD, especially for patients with SHPT (2, 3, 6, 29). Current evidence supports that improvement in 25(OH)D is associated with a decline in parathyroid hormone (PTH) levels (30). However, the association between 25(OH)D and kidney function has been controversial. Studies have shown that 25(OH)D has no protective effect on kidney function, and even has the opposite effect (7, 8). There were even case reports of acute kidney failure caused by the use of vitamin D drops (31). Teumer et al. (32) conducted a Mendelian randomization study and indicated the negative association between circulating vitamin D metabolite levels and eGFR. They also suggested that reduced kidney function can result in reduced serum vitamin D levels, which could cover up the effect of high vitamin D levels on low eGFR. Our results showed a consistent trend, which suggested a protective effect of low vitamin D levels on kidney function. Each unit of decreased serum 25(OH)D concentrations predicted 0.453 ml/min/1.73 m^2^ (95% CI: 0.426, 0.48, *p* < 0.0001) higher eGFR. The results remained stable after adjustment for sex, age, ethnicity, ratio of family income to poverty, education level, BMI, alcohol consumption, smoking history, vitamin D supplements, protein intake, serum albumin, heart disease, stroke, hypertension, and diabetes. In addition, our study was based on the general adult population in the USA. Compared with clinic-based studies, our results are more universally applicable and can more truly reflect the relationship between vitamin D and kidney function. However, the level of vitamin D is not the lower the better. The study of Jhee et al. (27) found that severe vitamin D deficiency was associated with renal hyperfiltration (RHF). Hence, vitamin D supplementation should be given cautiously for patients with decreased kidney function, and serum 25(OH)D concentrations should be detected regularly. At present, vitamin D supplement is mainly used to control secondary hyperparathyroidism and regulate bone metabolism disturbance and calcium and phosphorus metabolism disturbance caused by CKD. It is hoped that in the future, researchers could find a substitute for vitamin D supplement to improve the above symptoms and reduce the damage to kidney function caused by therapeutic drugs. For healthy people, it is not recommended to supplement vitamin D without indication. It is also a research direction in the future to find the appropriate serum 25(OH)D concentrations for different populations.

Patients with CKD have a higher risk of CVD and diabetes (33, 34). Metabolic syndrome is a predictor of CVD and diabetes (24). Referring to the definition of metabolic syndrome (Table 3) proposed by the National Cholesterol Education Program's Adult Treatment Panel III report (ATP III) (24), participants with severely decreased kidney function met the definition criteria in terms of waist circumference, systolic blood pressure, triglycerides, and blood glucose. This result was consistent with the expected trend. However, the mean levels of the components of metabolic syndrome by serum 25(OH)D quartile were not up to the defined criteria. Although some studies have suggested an association between low vitamin D levels and metabolic syndrome, the evaluation method they used varied (18, 19), and there are few related clinical trials. Therefore, it is difficult for us to obtain a definite conclusion based on the current evidence. Another problem is that the current definition of metabolic syndrome is composed of five indicators. A more comprehensive index is needed to help researchers better explore and evaluate metabolic syndrome.

Our study showed the inverse association between kidney function represented by eGFR and IR, which was similar to previous studies (35–37). Combining the above associations between kidney function and metabolic syndrome, our study provided solid evidence that kidney function caused metabolic perturbations. This might explain why patients with CKD had a higher risk of CVD. The inverse association also existed between serum 25(OH)D concentrations and IR, which supported the studies of Szymczak-Pajor et al. (15) and Garbossa et al. (16). The difference between our study and their studies is the choice of study population. Their studies were based more on clinical patients, and ours was based on the population level. In addition, we adjusted diabetes status rather than excluding patients with diabetes during the analysis to make our results more generalized. Therefore, for patients with IR, we recommend the use of vitamin D supplements for improvement, but kidney function should be monitored regularly because CKD has a high prevalence in patients with diabetes (38). For patients with both CKD and diabetes, it is recommended to use vitamin D supplement cautiously after a comprehensive assessment of the patient's condition. The appropriate dose of vitamin D supplement for different populations needs to be further explored in the future.

One strength of this study is that we used a representative sample of the population of USA and included more than 36,000 samples. In addition, many covariates were adjusted in the analysis process to ensure that our results were applicable to a wide range of people. However, there were still some limitations in this study. Since this was a cross-sectional study, the evidence for causal inference was weak, which led to the directionality of association not being determined. Furthermore, due to the lack of data for direct measurement of GFR and insulin sensitivity, we cannot exclude the possibility that some of the associations observed in this study result from differences between estimated and true values. In addition, although some other confounding factors might influence the vitamin D level (such as milk intake), we did not include them due to coverage of already existing covariants or lack of data in NHANES cycles. Therefore, we could not adjust it in the process of analysis.

In conclusion, our results showed that the mean serum 25(OH)D concentrations were increased in participants with decreased kidney function and that serum 25(OH)D concentrations were negatively associated with kidney function. In addition, IR appears in the early stage of kidney dysfunction, and both serum 25(OH)D concentrations and kidney function were negatively associated with IR. Further studies are needed to find the appropriate serum 25(OH)D concentrations and the appropriate dose of vitamin D supplement for different populations. The underlying mechanism of these associations still needs more research, especially the negative association between serum 25(OH)D concentrations and kidney function.

## Data Availability Statement

Publicly available datasets were analyzed in this study. This data can be found here: cdc.gov/nchs/nhanes/index.htm.

## Ethics Statement

The studies involving human participants were reviewed and approved by National Center for Health Statistics. The patients/participants provided their written informed consent to participate in this study.

## Author Contributions

JG and YQ designed the research, analyzed the data, and wrote the paper. YL, JL, RL, YQ, and HD assisted in data analysis. LJ, ZQ, QY, and ZZ assisted in manuscript preparation. BS had primary responsibility for the final content. All authors read and approved the final manuscript.

## Funding

This work was financially sponsored by the State Key Research Program of China (Grant No. 2016YFC1103004, Grant No. 2016YFC1103003), the National Natural Science Foundation of China (Grant No. 82000702), the Science and Technology Achievement Transformation Fund of West China Hospital of Sichuan University (Grant No. CGZH19006), and the National Clinical Research Center for Geriatrics, West China Hospital, Sichuan University (Grant No. Z2018B10).

## Conflict of Interest

The authors declare that the research was conducted in the absence of any commercial or financial relationships that could be construed as a potential conflict of interest.

## Publisher's Note

All claims expressed in this article are solely those of the authors and do not necessarily represent those of their affiliated organizations, or those of the publisher, the editors and the reviewers. Any product that may be evaluated in this article, or claim that may be made by its manufacturer, is not guaranteed or endorsed by the publisher.
