# Supplementary figures and images for: Associations Between 25-Hydroxyvitamin D, Kidney Function, and Insulin Resistance Among Adults in the United States of America
Source: Front Nutr. 2022 Feb 15;8:716878. doi: 10.3389/fnut.2021.716878 (PMC8885514; doi:10.3389/fnut.2021.716878)

**Supplemental Figure 1.** NHANES 2000-2014 analytic sample flow chart.

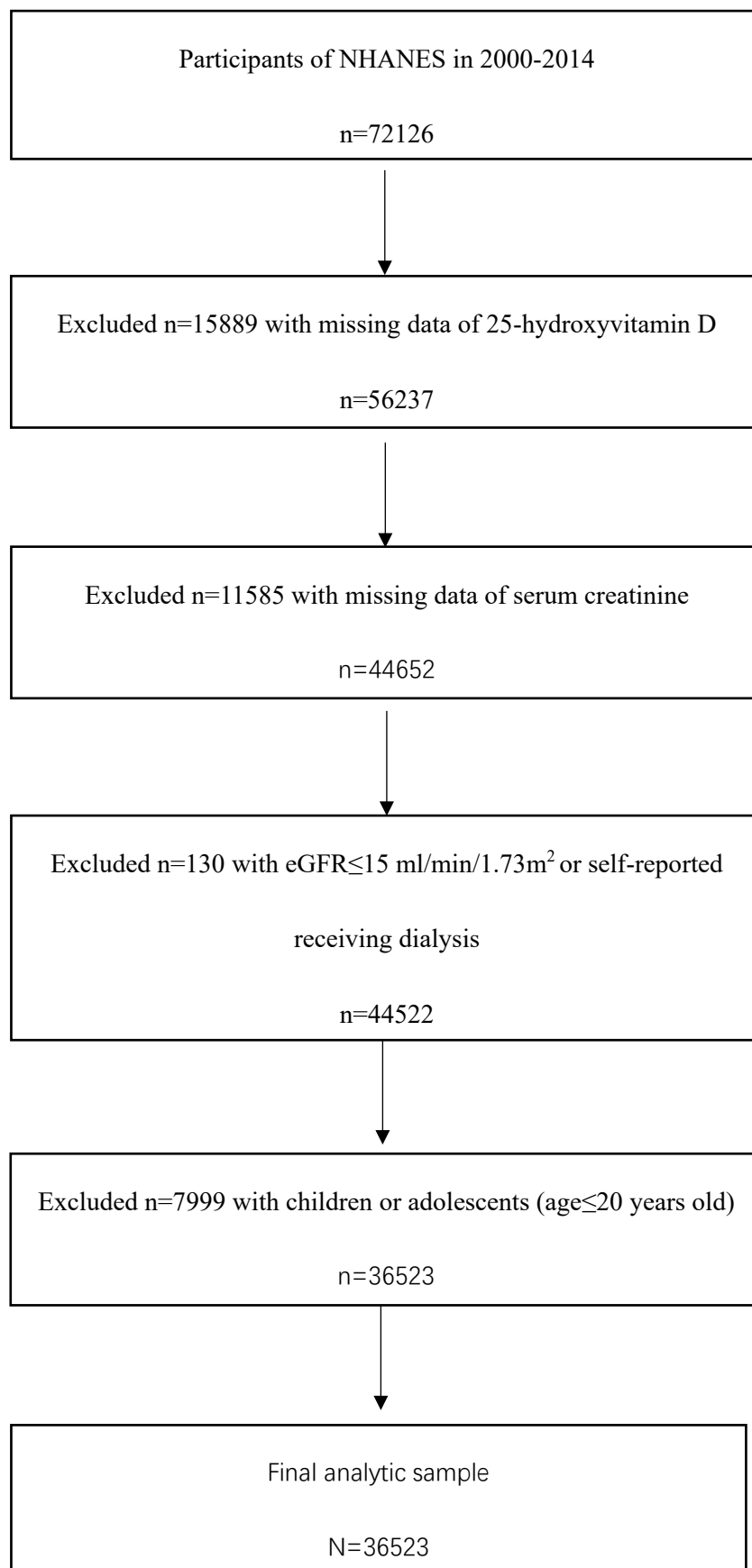

Supplement: Supplementary file 1 [file Image_1.pdf]
